# Collaboration among Registered Nurses and Licensed Practical Nurses: A Scoping Review of Practice Guidelines

**DOI:** 10.1155/2020/5057084

**Published:** 2020-06-02

**Authors:** Dawn Prentice, Jane Moore, Joanne Crawford, Sara Lankshear, Jacqueline Limoges

**Affiliations:** ^1^Department of Nursing, Faculty of Applied Health Sciences, Brock University, 1812 Sir Isaac Brock Way, St. Catharines, Ontario, Canada L2S 3A1; ^2^Health, Wellness and Sciences, Georgian College, Barrie, Ontario, Canada

## Abstract

Professional associations, nurse scholars, and practicing nurses suggest that intraprofessional collaboration between nurses is essential for the provision of quality patient care. However, there is a paucity of evidence describing collaboration among nurses, including the outcomes of collaboration to support these claims. The aim of this scoping review was to examine nursing practice guidelines that inform the registered nurse (RN) and registered/licensed practical nurse (R/LPN) collaborative practice in acute care, summarize and disseminate the findings, and identify gaps in the literature. Ten practice guidelines, all published in Canada, were included in the final scoping review. The findings indicate that many of the guidelines were not evidence informed, which was a major gap. Although the guidelines discussed the structures needed to support intraprofessional collaboration, and most of the guidelines mention that quality patient care is the desired outcome of intraprofessional collaboration, outcome indicators for measuring successful collaborative practice were missing in many of the guidelines. Conflict resolution is an important process component of collaborative practice; yet, it was only mentioned in a few of the guidelines. Future guidelines should be evidence informed and provide outcome indicators in order to measure if the collaborative practice is occurring in the practice setting.

## 1. Background

Over the past two decades, the health care system has undergone a significant transformation which has required that all team members work to their full scope of practice. In order to practice to full scope, registered nurses (RNs) and registered/licensed practical nurses (R/LPNs) are required to work together using a collaborative practice model of care to meet the needs of complex patients [1]. Collaboration among health care providers has long been regarded as a means for ensuring optimal quality patient care [2]. As such, the profession has developed guidance documents such as practice guidelines to support nurses to strengthen their collaborative practice skills.

Collaboration in the context of health care delivery is described as working together with one or more members of the health care team with each member making a unique contribution toward achieving a common goal [3]. Collaboration between team members from the same profession is referred to as intraprofessional collaboration [3], and among nurses, it is viewed as a relational process between colleagues who share common professional education, values, socialization, identity, and experience [4]. Engaging in collaborative practice is a professional expectation and is a required competency for all categories/designations of nurses in many countries [5–8].

The nursing profession in Canada is comprised of four different categories of nurses including registered nurses (RNs), registered/licensed practical nurses (R/LPNs), registered psychiatric nurses (RPNs), and nurse practitioners (NPs). The term LPN is used extensively across North America except in the province of Ontario, Canada, where LPNs are referred to as registered practical nurses (RPNs). The basic entry to practice educational requirements for RNs and R/LPNs varies across Canada. However, in most provinces, entry to practice for an R/LPN is a two-year college diploma program and RN entry to practice is a four-year baccalaureate program. All nurses must then successfully pass a registration examination in order to practice. While there are differences in educational preparation and scope of practice, the core values of nursing remain the same for both RNs and R/LPNs. Core values include providing safe, compassionate, competent, and ethical care; promoting health and well-being; promoting and respecting informed decision-making; preserving dignity; maintaining privacy and confidentiality; and being accountable [6, 9].

Registered nurses and R/LPNs have a long history of collaborative practice in rehabilitation and long-term care settings and more recently in acute care hospitals. Despite requirements for collaboration, studies show that there is tension between nurses working on teams [1]. Oelke et al. [10] noted that unclear role definitions and heavy workload pressures were barriers to effective teamwork. Kalisch and Begeny [11] reported that large team size, lack of familiarity among team members, team instability (high turnover), lack of a common purpose and goals, and clinical unit structure were all factors that inhibited high-performing nursing teamwork. Moore et al. [12] found that a lack of working to the full scope of practice, role ambiguity, age and generational differences, and interpersonal skills discouraged collaboration among RNs and R/LPNs. Undergraduate nursing education provided in silos and the lack of specific curriculum addressing intraprofessional practice were also cited as significant barriers [13, 14].

The Registered Nurses Association of Ontario (RNAO) [15] suggests that guidelines offer instruction on creating, enhancing, and sustaining collaborative relationships among nurses that promote high-quality patient care. They also provide guidance for nurses to engage in collaborative practice and reinforce professional and regulatory responsibilities to make ethical and safe decisions [15]. As such, resources such as collaborative practice guidelines have been developed to assist in improving collaboration among nurses. Given the complexity of patient care needs, increasing workloads and efforts to maintain quality patient care, there is a need to review existing evidence on nursing collaborative practice among RNs and R/LPNs.

The purpose of this scoping review was to examine the practice guidelines related to RN and R/LPN collaborative practice in acute care, summarize and disseminate the findings, and identify any gaps in the literature. This paper reports on findings related to nursing practice guidelines that emerged from the original scoping review [16].

## 2. Method

Arksey and O'Malley's [18] framework for scoping reviews was used for this study. Their framework encompasses five steps: (a) identifying the research question, (b) identifying studies relevant to the research questions, (c) selecting the studies, (d) charting the data, and (e) reporting the results. The main research question for the scoping review was as follows: *What is known from the existing guidelines about the structures, processes, and outcomes of RN and R/LPN collaboration in acute care settings?*

### 2.1. Search Strategy

The search strategy for the initial scoping review included all published and unpublished data including grey literature from 1990 to July 2017 in the following databases: OVID Medline, CINAHL, the Cochrane Library, ProQuest/Allied Health, APA PsycNET, OVID HealthSTAR, Web of Science Complete, and EMbase. In addition, key journals were hand searched. Nursing Association websites from across North America were also accessed for relevant literature. During this search, ten practice guidelines were identified. Practice guidelines offer nurses' instructions or a roadmap for enabling collaborative practice. Practice guidelines also address collaboration as a competency required for clinical practice, an important part of the nursing role that is required to provide safe and quality patient care. Since ten guidelines were identified and because they addressed collaboration in a unique manner, the research team decided that a separate review of these guidelines was warranted. Information regarding the initial scoping review has been previously published and focused solely on the studies reviewed [16].

### 2.2. Analysis

Donabedian's [19] quality framework that assesses structure, processes, and outcomes was used as a framework for analysis and reporting of the findings. For this review, structure encompassed factors that influence collaboration among RNs and R/LPNs such as role descriptions, staffing models, collaboration models/frameworks, education and training, policies, and regulations. Processes included factors that influence collaboration among RNs and R/LPNs such as communication, interpersonal skills, clinical competency (e.g., knowledge and technical skills), facilitators, and barriers. Outcomes of RN and R/LPN collaboration referred to the quality of patient care, satisfaction (patient or nurse), morbidity rates, mortality rates, near-miss/error/adverse events, nurse-recruitment and retention, absenteeism, satisfaction, conflict, and bullying. Two members of the researcher team independently reviewed the 10 guidelines using Donabedian's [19] framework, compared individual results, and used consensus decision-making when needed (Table 1).

## 3. Findings

The 10 documents retrieved from the original search represented practice guidelines from seven Canadian provinces, a staff mix decision-making framework published by the Canadian Nurses Association (CNA) [21], and two practice guidelines published by the Registered Nurses Association of Ontario [15, 24] with the latter being a revision of an earlier version. Publication dates for the documents ranged from 2003 to 2017.

Although all 10 documents were presented as guidelines, there was variability among the documents. The CNA [21] document was developed by a panel of registered nurses, registered/licensed practical nurses, registered psychiatric nurses, and unregulated care providers from across Canada and presents a framework for decision-makers for staffing decisions. Furthermore, the authors of the document assert that it is “a comprehensive and evidence-informed resource presenting a systematic approach to staff mix decision-making that can be used in all clinical practice settings” (p. 5). The RNAO guidelines [15, 24] were developed by a panel of experts including staff RNs and R/LPNs, practice leaders, nurse executives, and academic educators and based on a systematic review of evidence. The RNAO guidelines also provided recommendations for a healthy work environment based on the evidence. Prior to publication, the RNAO guidelines were reviewed by another panel of stakeholders. The seven remaining guidelines provided information or guiding principles to promote collaboration but did not state that they were based on available evidence.

Six out of seven provincial guidelines were coauthored by their respective RN and R/LPN associations/regulators. One practice guideline (Ontario) was authored by the provincial nurse regulator because at the time when the scoping review was conducted, the College of Nurses of Ontario was the only nursing regulatory body in Canada that represented both RNs and R/LPNs.

Six of the guidelines published by provincial regulators provided a definition of collaborative practice [3, 4, 17, 20, 22, 23] as did the two healthy work environments best practice guidelines [15, 24].

### 3.1. Structure

Six of the provincial nursing association/regulator documents discussed the roles of the RN and R/LPN, provided clarification about the different nursing roles, and outlined the responsibilities of each nursing role when working collaboratively [3, 4, 17, 20, 22, 23].

Five of the guidelines outlined the applicable legislative components for each category of nurses [4, 17, 20, 23]; however, Ontario was the only province where one legislation represents both RNs and R/LPNs practice [3]. Four guidelines specifically addressed the scope of practice for each category of nurses [3, 4, 17, 23]. In two provincial guidelines [17, 23], the R/LPN works under the direction of an RN or another medical practitioner, and in the Nova Scotia [4] and Ontario guidelines [3], R/LPNs are expected to consult with an RN if the client becomes unstable and thus requires more complex care. In one guideline [17], the R/LPN is able to work as a team member only, whereas the RN can work as an independent practitioner or as a team member in all clinical settings. Although the legislation was defined in the CNA [21] document, the actions and responsibilities of each nurse or caregiver were not well described.

The difference in educational qualifications for each category of nurses was addressed in the provincial guidelines from British Columbia, New Brunswick, Nova Scotia, and Prince Edward Island. A baccalaureate degree in nursing was required for entry to practice for registered nurses in all provincial guidelines noted above. Although the R/LPNs' entry to practice requirements varied slightly, all four guidelines noted that program completion from an approved practical nursing school and successful completion of the registration exam is necessary for entry to practice. Only one guideline from the province of British Columbia stipulated a program length of 12 months for R/LPNs [22].

While not specifically outlining the roles of the RNs and R/LPNs, the RNAO's [15, 24] best practice guidelines for healthy work environments recommended that all nursing regulatory bodies work together and cover the roles and responsibilities of different health care providers and the educational preparation for each role and outline the scope of practice for each role.

The utilization of the different categories of nurses in the practice settings was discussed in six of the documents [3, 4, 17, 20, 21, 23]. However, the CNA [21] provided the most comprehensive framework for making staffing utilization decisions. In their Staff Decision-Making Framework, the CNA [21] provides nursing administrators with a list of factors to consider at the client, staff, and organization levels, when determining staff utilization as well as outcome indicators for each level. Two provincial nursing regulator documents [3, 4] provided a framework for the utilization of RNs and R/LPNs which considered the client, nurse, and environment when making staff utilization decisions.

Both editions of the RNAO [15, 24] healthy workplace best practice guideline provided a comprehensive, evidence-based model that examined factors that influenced healthy work environments, specifically a framework to promote collaborative practice among nurses. This framework consists of three major areas: physical/structural/policy components, cognitive/psycho/socio/cultural components, and professional/occupational components. Moreover, the framework examined these factors at a micro (individual), meso (organizational), and macro (external factors) level. The second edition of the RNAO healthy workplace environment best practice guideline [15] also noted that organizations may promote collaborative practice by implementing shared governance models and supporting all staff to work to the full scope of practice. Another suggestion was to develop competencies for intraprofessional practice that are linked to performance appraisals [15].

### 3.2. Process

After a review of the ten documents, it was noted that guiding principles for effective collaboration were present in five of the provincial guidelines [3, 4, 22, 23, 25].

The need for effective communication among nurses was noted in nine of the 10 documents [3, 4, 15, 17, 20–24], and the requirement for nurses to respect each other during the collaborative process was also mentioned in nine guidelines [3, 4, 15, 17, 20–24]. Consultation with other nurses, when deemed necessary, was discussed in six of the guidelines as an essential component of collaboration, [4, 17, 20, 21, 23, 25]. Surprisingly, conflict resolution was only addressed in three of the guidelines [15, 21, 24].

The RNAO [15, 24] healthy workplace guidelines provided recommendations to promote a collaborative workplace. One example of a recommendation at the individual level is that a nurse must be willing to communicate with others and value teamwork. At the organizational level, recommendations included the promotion of respectful communication, articulation of the scope of practice of each nurse, and development of clear processes that promote collaboration including conflict management resolution structures [15, 24]. The RNAO guidelines [15, 24] also recommended that management support teamwork with the resources to promote collaborative practice; nurse managers should model effective team behaviors [15] and also have nurses mentor students to create supportive learning environments that are collaborative in nature [15]. Several guidelines addressed clinical competency as essential to collaboration [3, 4, 17, 20–23, 25]. Clinical competency refers to the theoretical knowledge and technical skills nurses need to provide safe patient care. These clinical competencies are important components as they contribute to the development and maintenance of mutual trust and respect and are critical requirements of successful collaborative practice.

### 3.3. Outcome

All 10 papers referred to *quality client outcomes or safe patient care* as important outcomes of collaborative practice. The Canadian Nurses Association [21] in their staff mix decision-making framework provided detailed outcomes for the client (e.g., safe quality patient care, satisfaction, morbidity, and mortality); the nursing staff (e.g., job satisfaction, decreased turnover, and decreased absenteeism); and the organization (e.g., safe patient care, quality of work environment, and human resources costs). The RNAO's [24] healthy workplace guideline noted that healthy work environments benefit the client with respect to quality patient care and patient satisfaction, job satisfaction for nurses, improvements in patient outcomes, and reduction in absenteeism and costs from adverse patient outcomes for organizations. The RNAO also cautions that team outcomes need to be measured and monitored.

The analysis of the 10 intraprofessional guidelines using Donabedian's framework demonstrated considerable variability in both content and format of the guidelines. The structures involved in collaboration were discussed in all guidelines, while the processes involved in intraprofessional communication were somewhat covered by mentioning the need for respect and effective communication. However, the outcomes of intraprofessional collaboration were only comprehensively addressed in three of the guidelines. These findings will be explored further in the next section.

## 4. Discussion

The purpose of this scoping review was to examine practice guidelines related to RN and R/LPN collaborative practice in acute care, summarize and disseminate the findings, and identify gaps in the literature. Surprisingly, all guidelines found in the initial search were Canadian based. One could posit that this may be due to Canada's national health care system [26], which is taxpayer funded. Due to rising health care expenditures, there is a constant need to scrutinize costs and ensure that the right nurse is taking care of the right client at the right time. Alternatively, in many jurisdictions in Canada, the R/LPN is an autonomous nursing position and collaborates with the RN but does not work under the direction of an RN (e.g., [3, 27]) which may differ from other countries.

A noticeable gap was that only three of the ten guidelines [15, 21, 24] were based on available evidence. In the case of the remaining seven provincial guidelines, there was limited use of supporting evidence or literature to substantiate their recommendations or their guiding principles for ensuring a collaborative practice setting.

The guidelines discussed many of the *structures* required to support a collaborative work environment. However, to promote a collaborative work environment, there needs to be sufficient resources in terms of nurse staffing levels and an articulated nursing care delivery model to ensure an appropriately skilled workforce [28, 29]. This has policy implications for organizations because although there are guidelines to promote collaboration, each organization must value collaborative practice and incorporate it into their patient care philosophy first and then ensure that the workplace has sufficient nursing resources in place to meet patient needs and provide a collaborative work environment. As fiscal constraints continue in the health care setting, maximizing nursing resources including having all nurses working to their full scope of practice will be essential.

Although most of the guidelines mention the need for respectful and effective communication, another gap is that many do not detail what effective and respectful communication should look like. Given that nursing is a relational profession, specifically detailing the expected competencies related to effective communication is important [30]. Moreover, only three of the guidelines addressed conflict resolution, an essential component of collaboration. Conflict is not always negative, it can be constructive, and as such addressing conflict can enable improved decision-making, can improve individual nurse and team performance, and can result in the development of a better approach to patient care [31–33]. The authors recommend that all collaborative practice guidelines should include conflict resolution as a component.

It is not surprising that all 10 guidelines at least mentioned the need for collaboration to ensure the best possible patient outcomes, since patients who are cared for by nurses who work collaboratively on teams have reportedly more improved safety outcomes [34]. Moreover, the quality of patient care and improved outcomes are better in settings where nurses are engaged and satisfied with the workplace environment [35–37]. However, only three of the documents [15, 21, 24] provided more robust outcomes that could be measured as a result of intraprofessional collaborative practice. Providing structures and processes for collaboration as well as outcome measures to ensure collaboration is recommended. High functioning teams are more likely to have reduced incidence of errors and missed nursing care [38, 39].

As evidenced by the findings of this scoping review, there are guidelines from nursing regulatory bodies that outline structures and some processes for intraprofessional collaboration. However, the reality is that collaboration in the workplace is not always happening. Some studies have shown that barriers to collaboration are nurse based. Collaboration must be valued by the nurse, and it is essential that the nurse has the interpersonal skills to engage in collaboration [12, 40]. Yet not every nurse may possess the interpersonal skills or the desire to collaborate. From an organizational perspective, the RNAO [15] suggests that each nurse should be evaluated based on their collaboration skills. This may necessitate the need to develop tools that can be added to performance appraisals to measure individual nurse's collaboration in the workplace. Tying performance to collaboration may also have an impact on the individual's perception of its value to nursing practice, workplace satisfaction, and patient outcomes. Essentially, the data from performance appraisals could be used by the organization for quality assurance to assess how well they are doing and where additional training and education are required. Further research would be important to examine if the use of performance appraisal tools is effective in enhancing collaborative practice longitudinally.

Clear policies and position descriptions must be available for all categories of nurses in hospitals, so they can understand each other's role and scope of practice. Given that scope of practice and roles of various nurses may not be discussed in nursing curricula, another potential solution is for policymakers in hospitals to include this as part of orientation for new nursing employees.

Finally, some of the guidelines were published over ten years ago, one in 2003 [20] and the other in 2009 [16]. Furthermore, other guidelines are at least five years old. Given the changes in the scope of practice for the R/LPN position in many provinces, there is a need to update and revise the guidelines to ensure that the practice standards reflect the current state of the nursing positions in acute care settings.

### 4.1. Limitations

There are several limitations to our scoping review. We limited our search to full-text and English language only documents; therefore, some guidelines may have been missed. In addition, the reviewed guidelines and practice standards were developed by nursing regulatory bodies and professional organizations which may not represent actual collaboration processes in acute care settings. While we searched North American nursing websites, we did not search internationally and therefore may have missed collaboration guidelines from other countries.

## 5. Conclusion

Intraprofessional collaboration is an essential element of a healthy workplace environment. Although there are written guidelines for *how* to collaborate and utilize nursing resources effectively, they need to be evidence informed and provide resources for nurses who work in the clinical setting. Findings from this scoping review of practice guidelines indicate that intraprofessional collaboration is the responsibility of all nurses at the bedside, at the organizational level, and at the policymaking level. Since the conclusion of the scoping review, the province of British Columbia and the province of Nova Scotia have merged all nursing professionals under one college which will further collaborative efforts. However, this does not guarantee that any changes will be seen in clinical settings. The authors recognize that while these guidelines were published in Canada, they are relevant to other countries where different categories of nurses are employed and there is a need to promote effective intraprofessional collaboration.

## Figures and Tables

**Table 1:** Summary of findings.

Lead author/year country	Title of guideline	Aim	Category: structure (S) process (P) outcome (O)
Alberta Association of Registered Nurses, College of Licensed Practical Nurses (CLPNA), and the Registered Psychiatric Nurses Association of Alberta (RPNAA) [20], Canada	Collaborative Nursing Practice in Alberta	To describe collaborative practice for the three categories of nurses in Alberta and the responsibilities of each nurse's role (RN, registered psychiatric nurse & LPN)	S, P, O
Association of Registered Nurses of Prince Edward Island (ARNPEI), the Licensed Practical Nurses Association of Prince Edward Island (LPNA), and the Prince Edward Island Health Sector Council (PEIHSC) [17], Canada	Exemplary Care: RNs and LPNs Working Together	To clarify the roles of the RN and LPN in clinical practice	S, P, O
Canadian Nurses Association [21], Canada	Staff Decision-Making Framework for Quality Nursing Care	Provide a staff-decision-making framework about staff mix	P, O
College of Nurses of Ontario [3], Canada	Practice Guideline RN and RPN Practice: The Client, the Nurse, and the Environment	To assist nurses, employers, and others make decisions about the utilization of nurses in the provision of care	S, P, O
College of Registered Nurses of BC, College of Registered Psychiatric Nurses of BC, and the College of Licensed Practical Nurses of BC [22], Canada	Collaborative Nursing Practice in BC Nurses Working Together for Quality Nursing Care	To provide support for collaborative nursing practice for the three categories of nurses in BC	S, P, O
College of Registered Nurses of Nova Scotia and the College of Licensed Practical Nurses of Nova Scotia [4], Canada	Guidelines for Effective Utilization of RNs and LPNs in a Collaborative Practice Environment	To provide information for RNs and LPNs regarding their own and each other's roles To assist managers in making decisions for care assignments	S, P, O
Nurses Association of New Brunswick and Association of New Brunswick Licensed Practical Nurses [23], Canada	Guidelines for Intra-Professional Collaboration Registered Nurses and Licensed Practical Nurses Working Together	To clarify the roles of the RNs and LPNs and assist employers in making decisions about effective utilization of nursing resources	S, P, O
Registered Nurses Association of Ontario [15], Canada	Healthy Work Environments Best Practice Guideline and Intra-Professional Collaborative Practice among Nurses 2^nd^ edition	Describe intraprofessional collaboration among nurses	S, P, O
Registered Nurses Association of Ontario [24], Canada	Healthy Work Environments Best Practice Guideline and Collaborative Practice among Nursing Teams	Focus was on developing collaborative practice among nurses	S, P, O
The Association of Registered Nurses of Newfoundland and Labrador (ARNNL) and the College for Licensed Practical Nurses of Newfoundland & Labrador (CLPNNL) [25], Canada	Collaborative Nursing Practice- Guiding Principles	To provide guiding principles to facilitate a collaborative practice environment	P, O
